# Chemical composition of *Lavatera thuringiaca* L. biomass ash after pre-sowing stimulation of seeds with He–Ne laser light

**DOI:** 10.1038/s41598-023-27836-5

**Published:** 2023-01-11

**Authors:** Małgorzata Budzeń, Grzegorz Zając, Agnieszka Sujak, Joanna Szyszlak-Bargłowicz, Marcin Kafarski

**Affiliations:** 1 Institute of Agrophysics, Polish Academy of Sciences, Doświadczalna 4, 20-290 Lublin, Poland; 2 Department of Power Engineering and Transportation, Faculty of Production Engineering, University of Life Sciences in Lublin, Głęboka 28, 20-612 Lublin, Poland; 3 Department of Biosystem Engineering, Faculty of Environmental Engineering and Mechanical Engineering, Poznań University of Life Sciences, Wojska Polskiego 50, 60-627 Poznań, Poland

**Keywords:** Biofuels, Solid biofuels

## Abstract

The article presents the effect of pre-sowing *Lavatera thuringiaca* L. seeds stimulation with He–Ne laser light on the chemical composition (P, S, K, Ca, Mn, Fe, Ni, Cu and Zn content) of ash obtained after combustion of shoots of different ages. Results varied, but it was confirmed that pre-sowing exposure of seeds to this physical factor for 10-min has the most pronounced effect on K, Cu and Mn content increase (6%, 20%, 31% increase respectively) in the ash after the first year of vegetation as well as on Cu, K and Zn content increase (9%, 19%, 22% increase respectively) after the second year of vegetation. However, 30-min stimulation significantly increases Ca (8%), Mn (20%) and Fe (72%) content in ash after the second year as well as results in ash richer in Ca (22%), P (48%), K (70%) and Zn (95%) after the third year of *Lavatera* vegetation. The pre-sowing application of He–Ne laser light depending on the time of stimulation can intensify the content of preferable macro- and microelement groups in *Lavatera* ash, in respective cultivation years. It can be an innovative method of biomass ash enhancement and its more effective use in agriculture as commercial fertilizers substitute.

## Introduction

The use of biomass for heat and electricity is increasing in many countries^1^, however as a result large amounts of ash are generated^2^. Biomass ash is the solid residue that accumulates after the thermal combustion of plant biomass and contains a variety of macro- and micronutrients. An essentials condition for soil to be fertile is a sufficiently large stock of mineral nutrients which can be assimilated by plants. Mineral nutrients include elements that plants need in large quantities such as nitrogen, phosphorus, potassium, sulphur, calcium and magnesium as well as elements that plants require in smaller amounts such as iron, manganese, copper, zinc and nickel^3–5^. Various plant species may require nutrients in different concentrations, proportions or chemical forms for effective absorption^3^. Nutrient element deficiency contributes to lower plant photosynthetic efficiency and reduced plant biomass growth. Nitrogen, phosphorus, potassium and other nutritional elements play an important role in the formation of chlorophyll, nucleotides, phosphatides, alkaloids as well as in the formation of many enzymes, hormones and vitamins necessary for obtaining optimum yield^3,6,7^. One of elements sources can be ash obtained from biomass combustion. Ash contains minerals in the form of inorganic compounds and organometallic complexes (SiO_2_, CaO, K_2_O, P_2_O_5_, Al_2_O_3_, MgO, Fe_2_O_3_, SO_3_, Na_2_O, TiO_2_)^8^. Biomass ash can be used as fertilizer^9–14^ because it improves the physicochemical properties of soil^15,16^. Ash from plant biomass can be a good source of phosphorus comparable to commercial phosphorus fertilizer that is well absorbed by plants. It can be used as an effective source of P fertilisation on clayey sand^17,18^. A study by Bilenda and Meller^19^ showed a positive effect of ash application on soil reaction formation and sorption properties. Biomass ash contributed to an increase in the content of available forms of P, K and Mg^19^. Zapałowska et al.^20^ report that the application of biomass ash at a rate of 12.8 t ha^−1^ increased the potassium content of *Jerusalem artichoke* plants to 5.63 g K kg^−1^. Szpunar-Krok et al.^21^ found that application of biomass ash fertilizer increases potato tuber yields and resistance to mechanical damage under quasi-static loads. The use of biomass ash as a fertilizer may be a sustainable strategy to maintain the availability of elements including phosphorus in soils^11^ thus improving their agricultural suitability. The chemical composition of ash obtained from biomass depends primarily on the type of combusted biomass^8,22,23^.

Thuringian mallow (*Lavatera thuringiaca* L.) is a perennial plant of the mallow family (*Malvaceae*). It occurs naturally in a wide belt extending from the Adriatic Sea to central Siberia, through central Europe and southern Scandinavia^24^. *Lavatera* is a plant with agricultural potential. This perennial can be used for energy purposes^25,26^. Thuringian Mallow plants achieve remarkable size and have many well branched stems. *Lavatera* can give 22.7–27.1 t D.M. per 1 ha (in the second and third years of vegetation at two cuts per year) and yielded better than e.g. *Helianthus annuus* L. (15.6–16.1 t D.M. per 1 ha)^25^. The *Lavatera* biomass has comparable energy parameters (calorific value, C, H, N, S, fixed carbon, volatile matter and ash content) to typical energy crops as Miscanthus, Virginia mallow, Jerusalem artichoke or basket willow^26^. After harvest *Lavatera* biomass yield can be directly burned in a domestic stove. Moreover it is a good melliferous plant and it can also be used for forage and fibre^25,27–30^. Due to high flavonoid content its seeds and flowers can be used for medicinal purposes^31–36^. Despite *Lavatera* being an agriculturally useful plant, it germinates poorly. From literature reports, it is known that in order to improve the seeds quality, physical factors such as electric field, magnetic field, laser, or plasma are used. The effect depends on the plant species as well as the parameters of the physical factor used, i.e. surface power density, wavelength, time of exposure to the factor, dielectric permittivity of seeds. Therefore, we conducted a series of laboratory experiments, where we tested the effects of these several different physical factors on the germination process. In preliminary tests, the laser performed best, subsequently field experiment was designed and this factor was used for pre-sowing stimulation of *Lavatera* seeds.

The presented results are a part of larger studies on He–Ne laser stimulation of *Lavatera thuringiaca* L. seeds. The previous studies found that the exposition of mallow seeds to He–Ne laser light have a beneficial effect on their germination^37^, field emergence capacity and biometric features^38^. The effect of plasma on the seeds germination energy and capacity was also investigated and an increase in analysed parameters was found^39,40^. In the paper by Budzeń et al.^26^ it was proved that the application of pre-sowing He–Ne laser light stimulation affects the energy parameters of *Lavatera* biomass, namely the combustion heat and calorific value increase of the grown shoots. The results showed most pronounced effect—nearly double increase in dry matter yields after pre-sowing stimulation of seeds for 30 min in case of 2-year plants^26^. The previous research have proven that He–Ne laser light can be used as an environmentally safe physical factor affecting the process of production of *Lavatera thuringiaca* L. biomass of higher utility value. It can be an important issue for bioenergy production as well as for the agricultural sector.

The formation of innovation and competitiveness of various branches of production at the field (plantation) level is one of the determinants of the directions of agricultural research. Stimulation with He–Ne laser light can be one of the innovative methods contribute to improving agricultural yields. In agricultural sciences, competitiveness is considered as an effort to reduce production costs and improve the efficiency of the use of production potential. *Lavatera* is a competitive plant species due to a potentially lower cost of the plantation maintenance resulting from low habitats and fertilizers demand. It is a multi-year (perennial) plant, which implies the increasing yields at reduced costs of seed material. The results of agricultural research have an important impact on the formation of competitiveness. The publication of research data makes it possible to effectively transfer the results of scientific research to agricultural practice. The scientific research presented in the article is directed at promoting agricultural innovation and competitiveness in *Lavatera* cultivation.

The aim of the study is to determine whether the pre-sowing stimulation of *Lavatera thuringiaca* L. seeds with He–Ne laser light can affect the chemical composition of ash after the combustion of the harvested biomass shoots. Understanding the relationship between irradiation times and energetic properties of biomass as well as studying the fertilising properties of ash by determining the content of macro- and microelements (P, K, S, Ca, Mn, Fe, Ni, Cu, Zn) will allow effective use of biomass of this plant in accordance with the principles of sustainable development. The application of ash for fertilisation is an opportunity to make optimal use of its value in the aspect of environmental protection.

## Materials and methods

The research material consisted of samples of 1-, 2- and 3-year old (years marked as I–III) *Lavatera* ash obtained during the combustion of biomass shoots after a 3-year field experiment. Representative samples from every experimental combination in four replicates n = 4 were collected and analyzed. All institutional, national and international guidelines and legislation were adhered to in the production of this study. Seeds of *Lavatera thuringiaca* L. Uleko cv. were obtained from the Plant Breeding and Acclimatization Institute (IHAR)—National Research Institute. Before sowing, the seeds of *Lavatera* were treated with He–Ne laser light of the following parameters: power density 6 mW cm^−2^, wavelength λ = 632.8 nm and exposure time 0–Control (C), 1, 5, 10, 15 and 30 min (L1, L5, L10, L15, L30).

The field experiment was conducted in Felin farm (51° 13′ 21.9″ N, 22^o^ 37′ 55.85″ E) on lessive soil developed from loess formations (Haplic Luvisol Soil) classified as good wheat complex (Sandy Clay Loam—SCL, according to USDA classification). For each group (C, L1–L30) 100 seeds with four replications were sown on 1 m^2^ harvest area micro-plots randomly located. In each subsequent year of the experiment, another plots side by side on homogenous experimental field were seeded. All samples of biomass shoots for analysis were collected in the same year and dried in a KBC G-100/250 oven (Premed, Poland) at 105 ± 2 °C for 24 h.

The effect of pre-sowing laser stimulation of *Lavatera* seeds on chemical composition of ash (macroelements—P, S, K, Ca and microelements—Mn, Fe, Ni, Cu, Zn content) was investigated for the cultivation year groups: C_I—L30_I, C_II—L30_II and C_III—L30_III respectively. The combinations after pre-sowing stimulation were analyzed against the respective control.

Determination of ash content was performed by the thermogravimetric method (TGA 701 LECO Corporation, Saint Joseph MI, USA) in accordance with EN ISO 18122 standards. For every experimental combination (C_I—L30_III) in triplicate the representative samples of *Lavatera* shoots biomass were milled with IKA A11 analytical mill (fractions sizes of 0.25–0.50 mm). A samples of m = 1 ± 0.2 g were placed in ceramic crucibles and then subjected to controlled heating from 25 to 900 °C. After heating treatment, all the samples were cooled before further testing.

The measurement of the chemical composition of *Lavatera* biomass ash was carried out using an XRF technique (XOS HD Maxine Analyzer). The content of respective elements P, S, K, Ca, Mn, Fe, Ni, Cu and Zn in all samples of *Lavatera* ash was determined.

In order to check the similarity within the studied groups, the Hierarchical Cluster Analysis—HCA (Ward’s Method, Euclidean Distance) was initially performed. The obtained results were analyzed with the use of analysis of variance—ANOVA. Normality of distributions was checked by the normal Q–Q plot; homogeneity of variance was checked by Bartlett’s test. To determine significant differences between the groups, pairwise comparison with the Tukey test was used. Analysis of the data was carried out in the program R i386 3.5.0 (GNU General Public License) at the significance level of α < 0.05. Graphs showing macro- and microelements concentration were made in the Grapher 18 (Golden Software LLC, USA).

## Results and discussion

### HCA of macroelements

**Figure 1:**
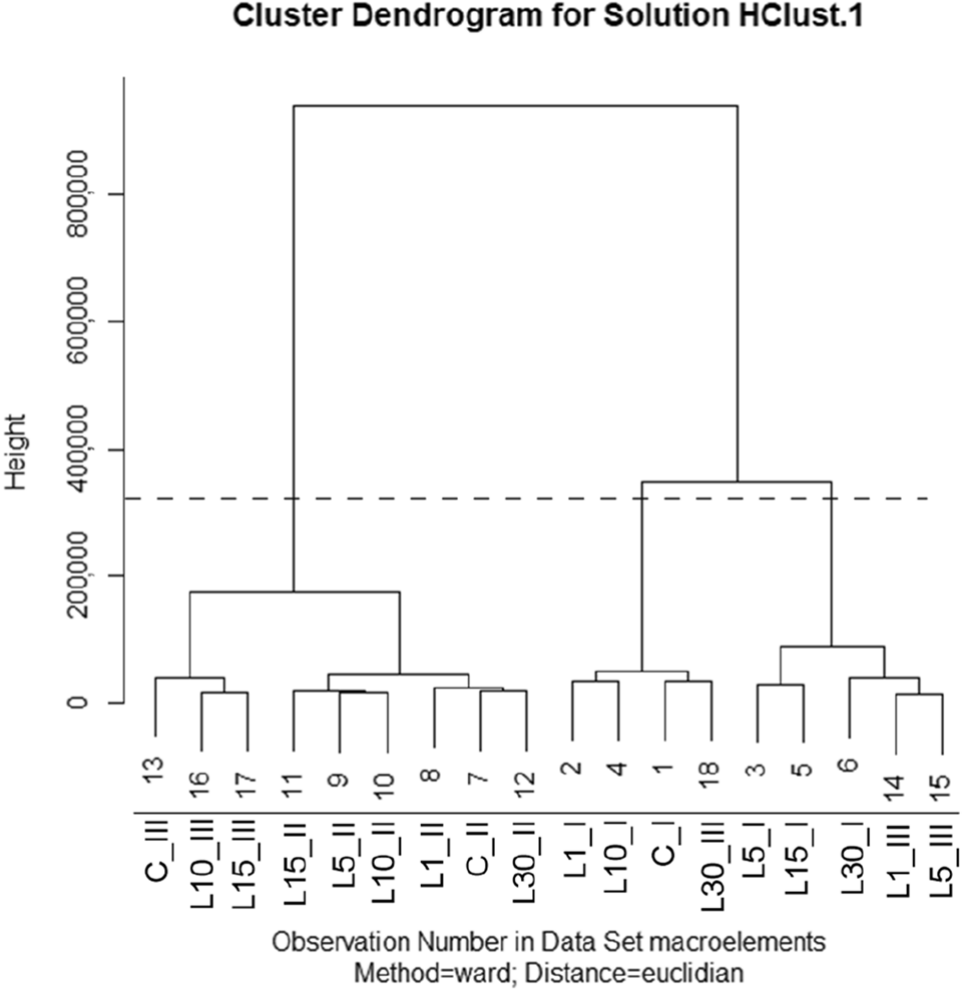
Dendrogram of the macroelements content (P, S, K, Ca) in the *Lavatera* ash after pre-sowing stimulation of seeds with He–Ne laser light, obtained from HCA.

During the hierarchical clustering analysis (Ward's Method, Euclidean Distance), three characteristic clusters of groups of macroelements were distinguished (Fig. 1).

The cut-off level was adopted by giving a 35% value of the last and at the same time the longest bond (100%) in dendrogram. The cutting off was performed at the height of 329,000.

For macroelements (P, S, K and Ca) the following clusters of groups were distinguished:

- I: C_I, L30_III, L1_I and L10_I,
- II: L1_III, L5_III, L30_I, L5_I and L15_I,
- III: C_II, L30_II, L1_II, L5_II, L10_II, L15_II, L10_III, L15_III and C_III (Fig. 1).

The first cluster is a group of 4 combinations—mainly 1-year combinations with stimulation times of 1, 10 min and 1-year control as well as a 3-year combination with a stimulation time of 30 min. The second cluster consists of 5 combinations—mostly 1-year combinations with stimulation times of 5, 15 and 30 min and 3-year combinations with stimulation times of 1 and 5 min. The third cluster consists of 9 combinations—all 2-year combinations and 3-year combinations with stimulation times of 10 and 15 min as well as a control.

### Analysis of P, S, K, Ca content in the *Lavatera* ash with different exposure times of He–Ne laser light (groups: C_I—L30_I, C_II—L30_II and C_III—L30_III)

As a complement to the HCA, a one-way ANOVA was performed to check whether there is an effect of the application of He–Ne laser light stimulation on the chemical composition of the *Lavatera* ash and whether there are statistically significant differences between the groups.

The ash content after the combustion of *Lavatera* shoots was in the range of 4.43–5.11% for 1-year, 3.44–4.18% for 2-year and 3.32–4.44% for 3-year combinations, respectively.

The results of macroelement determination in the investigated ash are presented in Table 1, for microelements in Table 2.

**Table 1:** Chemical composition (macroelements content) of *Lavatera* ash after pre-sowing stimulation of seeds with He–Ne laser light (ppm)—mean ± SD.

	Combination/Year	I	II	III
P	L1	93,973 ± 564 D	51,631 ± 240 C	61,080 ± 191 C
	L5	63,849 ± 330 B	46,535 ± 237 A	67,901 ± 912 E
	L10	91,189 ± 506 C	46,107 ± 368 A	55,544 ± 299 B
	L15	62,943 ± 502 AB	47,921 ± 254 B	52,671 ± 314 A
	L30	62,361 ± 389 A	51,574 ± 503 C	95,419 ± 690 F
	C	104,384 ± 131 E	48,427 ± 733 B	64,639 ± 56 D
	*	< 0.0000	< 0.0000	< 0.0000
S	L1	9184 ± 117 C	24,128 ± 58 C	10,874 ± 18 D
	L5	8195 ± 12 B	21,550 ± 62 B	10,771 ± 142 D
	L10	14,932 ± 125 D	29,343 ± 334 D	6346 ± 110 A
	L15	6860 ± 77 A	19,721 ± 165 A	7676 ± 59 B
	L30	7053 ± 35 A	30,312 ± 334 E	9449 ± 120 C
	C	26,480 ± 74 E	31,498 ± 333 F	13,322 ± 30 E
	*	< 0.0000	< 0.0000	< 0.0000
K	L1	324,040 ± 1277 D	91,256 ± 113 A	247,821 ± 1147 D
	L5	233,567 ± 1497 B	121,454 ± 207 D	247,291 ± 4128 D
	L10	355,970 ± 668 F	130,904 ± 1620 E	147,049 ± 1621 A
	L15	209,420 ± 560 A	119,464 ± 20 D	162,613 ± 1609 B
	L30	261,210 ± 681 C	100,918 ± 728 B	312,270 ± 1358 E
	C	336,502 ± 962 E	109,836 ± 1849 C	184,167 ± 869 C
	*	< 0.0000	< 0.0000	< 0.0000
Ca	L1	187,404 ± 824 D	191,903 ± 780 AB	196,506 ± 2289 C
	L5	150,310 ± 810 B	191,000 ± 436 A	185,888 ± 3877 B
	L10	191,723 ± 390 E	201,198 ± 3311 C	170,170 ± 2613 A
	L15	132,939 ± 2296 A	209,565 ± 58 D	175,591 ± 736 A
	L30	163,087 ± 516 C	211,036 ± 1137 D	225,564 ± 1262 D
	C	212,693 ± 689 F	195,549 ± 135 B	184,814 ± 1460 B
	*	< 0.0000	< 0.0000	< 0.0000

*C* control (untreated sample), *L1, L5, L10, L15, L30* samples from seeds subjected to pre-sowing stimulation with He–Ne laser light for 1, 5, 10, 15 and 30 min, respectively.

*Significance level, means with the same letter mean lack of significance difference at α < 0.05; capital letters indicate comparison of combinations: C_I—L30_I, C_II—L30_II and C_III—L30_III of *Lavatera* shoots.

**Table 2:** Chemical composition (microelements content) of *Lavatera* ash after pre-sowing stimulation of seeds with He–Ne laser light (ppm)—mean ± SD.

	Combination/Year	I	II	III
Mn	L1	626.33 ± 3.51 C	469.00 ± 2.00 AB	834.00 ± 7.21 BC
	L5	621.33 ± 3.21 C	457.00 ± 2.00 A	818.67 ± 17.47 B
	L10	851.67 ± 4.04 E	471.00 ± 8.00 B	698.33 ± 11.85 A
	L15	416.33 ± 11.37 A	495.00 ± 1.00 C	819.00 ± 3.46 B
	L30	524.33 ± 2.31 B	556.00 ± 5.57 D	862.67 ± 3.06 C
	C	650.00 ± 2.65 D	464.33 ± 5.13 AB	865.33 ± 20.55 C
	*	< 0.0000	< 0.0000	< 0.0000
Fe	L1	1294 ± 10.58 B	1937 ± 8.19 D	3570 ± 22.59 D
	L5	1165 ± 8.50 A	1379 ± 5.20 A	5767 ± 83.19 E
	L10	1805 ± 43.84 E	1893 ± 18.58 C	3310 ± 46.86 C
	L15	1950 ± 26.50 F	1880 ± 11.79 C	3224 ± 21.38 C
	L30	1707 ± 2.31 D	2842 ± 18.45 E	2925 ± 22.05 B
	C	1430 ± 5.51 C	1647 ± 0.58 B	2787 ± 8.08 A
	*	< 0.0000	< 0.0000	< 0.0000
Ni	L1	63.36 ± 1.29 B	106.00 ± 0.00 B	101.33 ± 0.57 C
	L5	49.61 ± 0.63 A	113.67 ± 0.58 C	132.67 ± 2.52 E
	L10	69.82 ± 1.20 C	101.15 ± 1.47 A	94.00 ± 1.93 B
	L15	69.68 ± 0.86 C	107.67 ± 0.58 B	68.11 ± 0.26 A
	L30	68.07 ± 0.55 C	117.00 ± 1.00 D	91.33 ± 1.45 B
	C	78.66 ± 0.24 D	116.33 ± 1.53 CD	108.67 ± 0.58 D
	*	< 0.0000	< 0.0000	< 0.0000
Cu	L1	192.33 ± 2.52 C	99.45 ± 1.36 A	165.00 ± 1.00 B
	L5	145.00 ± 1.00 A	131.67 ± 0.58 B	172.00 ± 2.65 C
	L10	258.00 ± 5.57 E	144.00 ± 1.00 C	180.00 ± 1.73 D
	L15	171.33 ± 2.52 B	142.67 ± 1.15 C	124.00 ± 1.73 A
	L30	152.00 ± 1.00 A	135.00 ± 1.00 B	181.33 ± 2.08 D
	C	215.67 ± 1.15 D	132.67 ± 2.08 B	184.00 ± 1.00 D
	*	< 0.0000	< 0.0000	< 0.0000
Zn	L1	446.67 ± 2.52 C	275.67 ± 0.58 B	338.33 ± 1.53 B
	L5	485.67 ± 4.62 D	239.33 ± 0.58 A	351.00 ± 5.29 C
	L10	427.67 ± 2.52 B	360.00 ± 3.00 F	336.33 ± 3.21 B
	L15	297.00 ± 5.57 A	312.67 ± 1.15 D	363.00 ± 2.65 D
	L30	491.33 ± 2.08 D	349.33 ± 2.08 E	565.67 ± 6.43 E
	C	527.00 ± 1.73 E	294.67 ± 1.55 C	289.67 ± 1.53 A
	*	< 0.0000	< 0.0000	< 0.0000

*C* control (untreated sample), *L1, L5, L10, L15, L30* samples from seeds subjected to pre-sowing stimulation with He–Ne laser light for 1, 5, 10, 15 and 30 min, respectively.

*Significance level, means with the same letter mean lack of significance difference at α < 0.05; capital letters indicate comparison of combinations: C_I—L30_I, C_II—L30_II and C_III—L30_III of *Lavatera* shoots.

#### Phosphorus

After the 1-year combinations had been analysed, the highest amount of phosphorus was recorded in C_I and the lowest in L30_I and. The smallest amount of P was observed in all samples treated with He–Ne laser light in relation to the control. In the case of 2-year combinations, the highest phosphorus content was recorded in L1_II and L30_II, and the lowest in L10_II. Statistically, L1_II and L30_II contained significantly more P and significantly less P was recorded in L5_II and L10_II relative to the control. After 3-year combinations had been analysed, L30_III had the highest amount of P and L15_III had the lowest amount of P. Furthermore, more P was observed in L5_III and L30_III relative to the control, and significantly less P was in the ash of L1_III, L10_III and L15_III (Table 1).

#### Sulphur

The combinations subjected to pre-sowing He–Ne laser light stimulation had less sulphur content compared to the control after the 3-years of cultivation.

#### Potassium

After the 1-year combinations had been analysed, a significantly higher content of potassium in the ash L10_I compared to the control was noted. In other combinations, K content in the ash was significantly lower in relation to the control. After the 2-year combinations had been compared, L5_II, L10_II and L15_II samples had significantly more K against the control, while L1_II and L30_II had less. In the case of the 3-year combinations, significantly more K in L1_III, L5_III and L30_III and significantly less in L10_III and L15_III in comparison to the control was observed. He–Ne laser light stimulation increased potassium content in the ash of the 1- and 2-year combinations of stimulation times of 10 min and of the 2-year combinations of exposure times of 5 and 15 min relative to the control. The 3-year combinations exposed to stimulation for 1, 5 and 30 min also had more potassium. In the other cases the trend was downward with respect to the control.

#### Calcium

In the 1-year combinations, a significantly lower calcium content was observed in the ash of all combinations subjected to pre-sowing exposure to laser light compared to the control. In the case of 2-year combinations, the L5_II had significantly less Ca and significantly more this element had L10_II, L15_II and L30_II as compared to the control. Among the 3-year combinations L1_III and L30_III had significantly more Ca in relation to the control and L10_III and L15_III had less Ca (Table 1).

### The macroelements content of *Lavatera* ash—graphs characteristics with respect to the HCA

**Figure 2:**
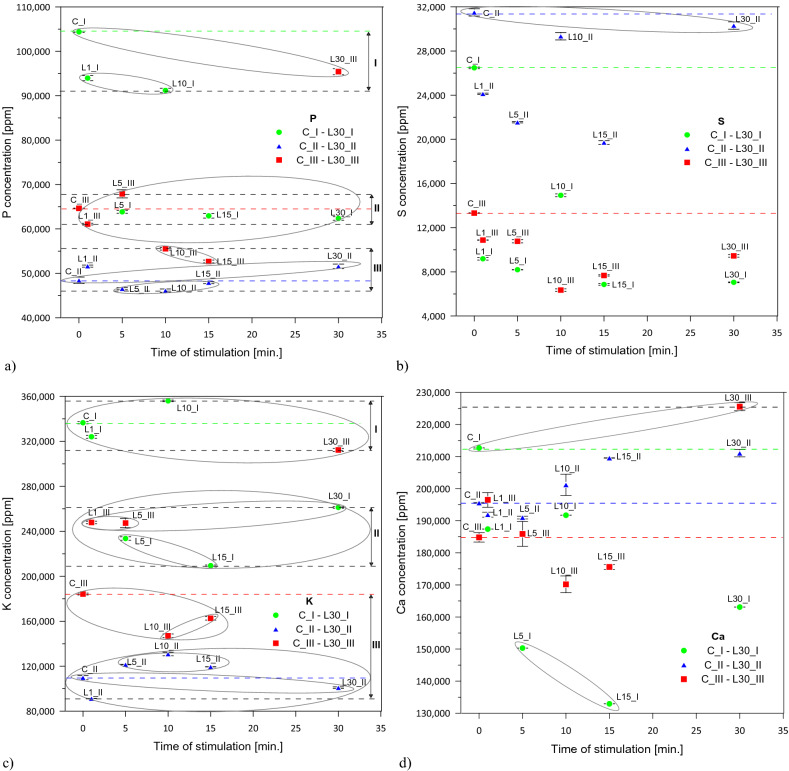
The macroelements content of *Lavatera* ash after pre-sowing stimulation of seeds with He–Ne laser light in 1-year (C_I—L30_I), 2-year (C_II—L30_II) and 3-year (C_III—L30_III) combinations: (**a**) P, (**b**) S, (**c**) K, (**d**) Ca [ppm]—mean ± SD.

In order to illustrate the results of the performed analyses, the content of macro- and microelements in the investigated combinations of 1-year (C_I—L30_I), 2-year (C_II—L30_II) and 3-year (C_III—L30_III) groups is shown in graphs (Figs. 2, 4).

Three characteristic clusters of groups for macroelements P and K derived from the hierarchical clustering analysis (Fig. 1) are presented in Fig. 2a and c.

The graph showing the clusters of groups for K and P (Fig. 2c) corresponds to the dendrogram model (Fig. 1), for P the only exception being C_III, which was found within the combinations from the third cluster. The groups of C_I and L30_III (for P, Ca and K) as well as L1_I and L10_I (for P and K) were highest in the hierarchy. In the case of sulphur and calcium, the clusters of groups were slightly more differentiated and deviating from the model of the presented dendrogram. For S, the 2-year combinations were the highest in the hierarchy, and among them C_II and L30_II (Fig. 2b). The 1-year combinations (except C_I) and the 3-year combinations formed a mixed cluster. For Ca, the 2-year combinations stimulated for 10, 15 and 30 min were as high as C_I and L30_III. The other groups L1_I, L10_I, C_II, L1_II, L5_II, C_III, L1_III and L5_III clustered similarly (Fig. 2d). An outlier group of combinations L5_I and L15_I, which ranked lowest in the hierarchy, was also observed (Fig. 2d).

The chemical composition of the *Lavatera* ash does not differ from other biomass types^13,41–43^ including mainly macroelements such as calcium, potassium, phosphorus, and sulphur.

The highest proportions of potassium, calcium, phosphorus and sulphur, respectively, in all 1-year combinations (C_I—L30_I) and also in L1_III, L5_III and L30_III were recorded. However, after the second year of plant vegetation, the highest proportions of calcium, and then potassium, phosphorus and sulphur, were observed. The same applied in the C_III, L10_III and L15_III. A similarity in the above-mentioned groups forming clusters illustrated in Fig. 1 was found.

**Figure 3:**
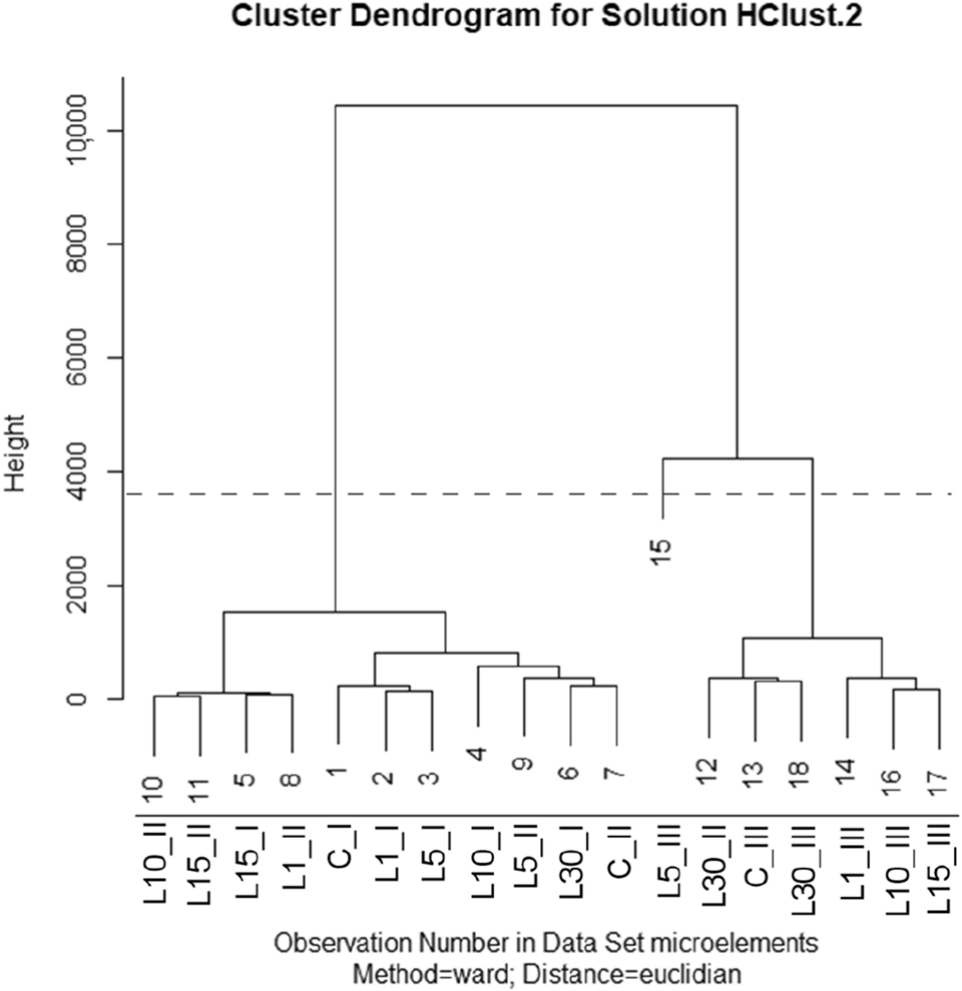
Dendrogram of the microelements content (Mn, Fe, Ni, Cu, Zn) of *Lavatera* ash after pre-sowing stimulation of seeds with He–Ne laser light, obtained from HCA.

The hierarchical clustering analysis (Ward's Method, Euclidean Distance) allowed, as in the case of macroelements, to distinguish three characteristic clusters, with the cut-off carried out at height 3675 (35% value of the longest bond). These clusters include:

- I: L10_II, L15_II, L15_I, L1_II, L1_1, L5_I, C_I, C_II, L30_I, L5_II and L10_I,
- II: C_III, L30_III, L30_II, L10_III, L15_III and L1_III,
- III: L5_III (Fig. 3).

The first cluster consists of 11 combinations—all of them 1-year and 2-year combinations (except 30_II). The second cluster consists of 6 combinations—the L30_II and the 3-year combinations excluding L5_III, which forms the third single-element cluster.

### HCA of microelements

See Fig. 3.

### Analysis of the Mn, Fe, Ni, Cu, Zn content in the *Lavatera* ash with different exposure times of seeds to He–Ne laser light (groups: C_I—L30_I, C_II—L30_II and C_III—L30_III)

#### Manganese

L10_I had significantly more manganese, while the L1_I, L5_I, L15_I and L30_I had less of this element with respect to C_I (Table 2). L15_II and L30_II had significantly more Mn than C_II. Other 2-year combinations did not differ in comparison to C_II. Significantly less manganese relative to the control was observed in L5_III, L10_III and L15_III, while other 2-year combinations showed no significant differences.

#### Iron

Analysis of the 1-year combinations showed that the L10_I, L15_I and L30_I had significantly more iron in comparison to C_I, while L1_I and L5_I had less. Also, L1_II, L10_II, L15_II and L30_II had significantly more iron when compared to C_II, while L5 had less iron content. In all stimulated 3-year combinations, significantly more Fe with respect to the control was observed. Laser light stimulation significantly increased iron content in all 3-year combinations when compared to the respective control.

#### Nickel

Comparing the 1-year combinations with the control statistically significantly lower amount of Ni in all combinations in compare to the control was noted. L1_II, L5_II, L10_II and L15_II had significantly less Ni when compared to the control. L1_III, 10_III, L15_III and L30_III also had a significantly lower amount of Ni with respect to C_III, however in L5_III the higher amount of Ni was recorded. In the 1-year combinations stimulated with laser light a significant decrease in nickel content when compared to the control was observed. This trend continued after the second year (except for combinations stimulated for 1 and 30 min—no differences when compared to the control) and after the third year of vegetation.

#### Copper

The L10_I had significantly more copper, while the other 1-year combinations had significantly less Cu when compared to the control. L10_II and L15_II had significantly more copper than C_II, while L1_II had less. In the case of L1_III, L5_III and L15_III significantly less copper with respect to C_III was noted. No statistically significant differences were observed in other combinations within this group.

#### Zinc

In all 1-year combinations which underwent the laser treatment, a significantly lower amount of Zn with respect to the control was recorded. L10_II, L15_II and L30_II had significantly more Zn compared to the control, while L1_II and L5_II had less. In all 3-year combinations, significantly more zinc when compared to the control was observed (Table 2). In the stimulated 1-year combinations, the zinc content in the *Lavarera* ash was decreasing when compared to the control. This trend was reversed in the 2-year combinations stimulated for 10, 15 and 30 min and in all stimulated combinations after the third year of vegetation.

### The microelements content of *Lavatera* ash—graphs characteristics with respect to the HCA

**Figure 4:**
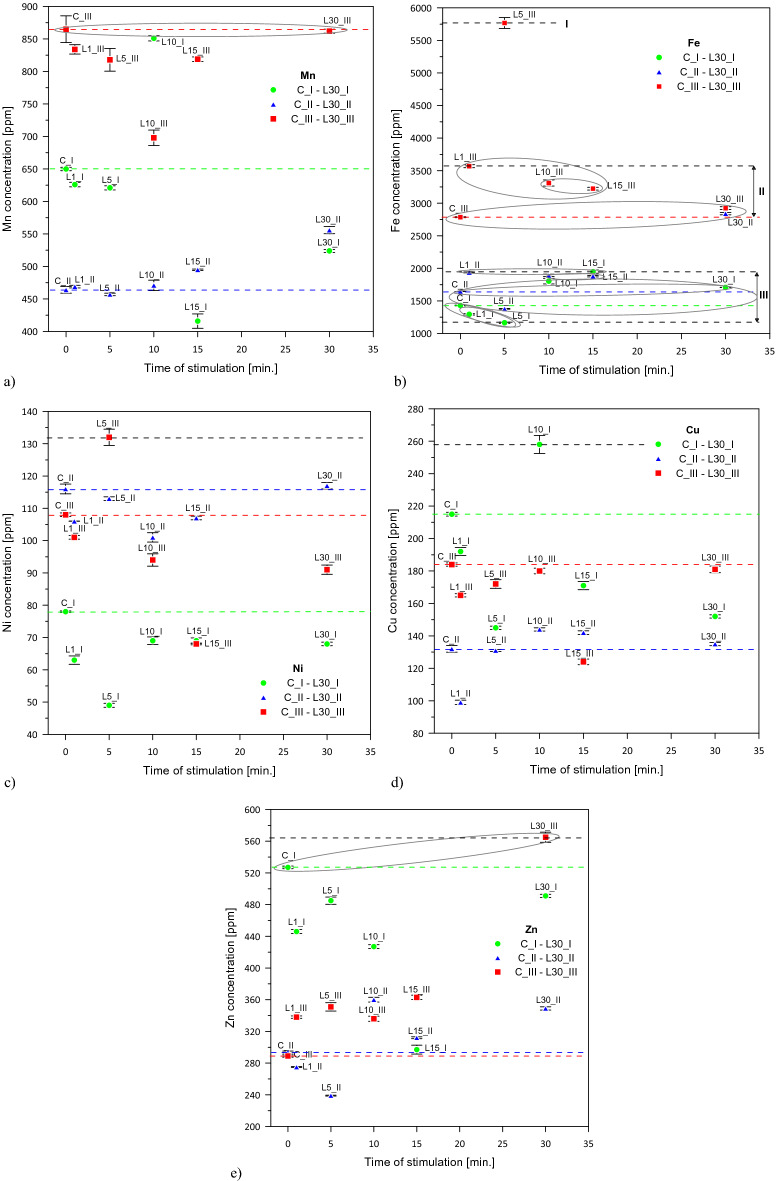
The microelements content of *Lavatera* ash after pre-sowing stimulation of seeds with He–Ne laser light for 1-year (C_I—L30_I), 2-year (C_II—L30_II) and 3-year (C_III—L30_III) combinations: (**a**) Mn, (**b**) Fe, (**c**) Ni, (**d**) Cu, (**e**) Zn [ppm]—mean ± SD.

On the basis of Fig. 3, characteristic clusters of groups for Fe were distinguished (Fig. 4b).

The 3-year combinations were the highest in the hierarchy and among them the combination L5_III. Analysing iron all 3-year stimulated combinations and L30_II were higher in the hierarchy than the 3-year control. Also for nickel, analogous to Fe, the combination L5_III had the highest position in the hierarchy (Fig. 4c).

For Mn, the 3-year combinations headed by the group C_III and L30_III and the combination L10_I were the highest in the hierarchy, similarly as in the case of iron, (Fig. 4a). Among the investigated combinations, the highest content of Cu was observed in L10_I (Fig. 4d). Characteristic groups C_I and L30_III similarly to macroelements P, K and Ca were also found in case of Zn (Fig. 4e). For Cu and Zn the 1-year and 3-year combinations were higher in the hierarchy when compared to the 2-year combinations.

In L10_II, L15_II and L30_II an increasing trend for Mn, Fe, Cu and Zn when compared to the control was observed (Fig. 4a,b,d,e).

In all analysed combinations within microelements, the highest proportions of iron, manganese, zinc, copper and nickel were found, respectively (the exception is L1_II, in which more nickel than copper was observed). Similar ranking of microelement content was found in herbaceous and woody biomass ash in the paper by Zając et al.^42^. In single cases, higher Ni than Cu content was also found. Romanowska-Duda et al.^13^ reported the highest proportions of iron, zinc, manganese and copper in the Jerusalem artichoke ash. On the other hand, the highest amount of Mn, Fe, Zn, Cu and Ni in biomass ash noted Magdziarz et al.^43^.

The use of stimulation times of 1 and 30 min results in ash richer in calcium and iron with the plants’ increasing age. These stimulation times also result in a significant increase in the phosphorus content of the 2-year combinations. The pre-sowing exposition of *Lavatera* seeds to He–Ne laser light for 10, 15 and 30 min increases the iron content in every year of vegetation and also the zinc content in the ash of the 2-year and 3-year combinations when compared to the respective controls. Summarise the chemical composition of *Lavatera* ash after a 3-year of vegetation in terms of pre-sowing stimulation of seeds with He–Ne laser light the effect was most pronounced for groups stimulated for 5, 10 and 30 min. A one and five minute stimulation gives better effects after the third year, a 10-min stimulation after the first two years, 15-min stimulation after the second year, while a 30-min stimulation after the second and third year of *Lavatera* vegetation.

The *Lavatera* biomass ash has comparable chemical composition to those of other energy crops and can be fertilizer source.

## Conclusions

The paper presents the effect of pre-sowing stimulation of *Lavatera thuringiaca* L. seeds with He–Ne laser light on the chemical composition of ash after combustion of biomass shoots. The content of respective elements in the ash of the 1- 2- and 3-year studied combinations varied. One-year cultivation of *Lavatera* can produce ash richer in potassium, calcium, phosphorus and sulphur, respectively, within macroelements, while 2-year cultivation can produce ash richer in calcium, potassium, phosphorus and sulphur. The use of pre-sowing stimulation of seeds with He–Ne laser light differentiates the proportions of ash elemental composition among macroelements after combustion of the 3-year *Lavatera* shoots. The application of shorter times of 1 and 5 min as well as the stimulation time of 30 min results in ash richer in potassium, while the 10 and 15 min stimulation times help obtain ash more abundant in calcium. Iron, manganese, zinc, copper and nickel are the major microelements found in the *Lavatera* ash. Pre-sowing exposure of seeds to He–Ne laser light for 10-min has the most pronounced effect on K, Cu and Mn content increase in the ash after the first year of vegetation as well as on Cu, K and Zn content increase after the second year of vegetation. However, 30-min stimulation significantly increases Ca, Mn and Fe content in ash after the second year as well as results in ash richer in Ca, P, K and Zn after the third year of *Lavatera* vegetation. In addition, the 5-min exposure of seeds to He–Ne laser light significantly increased the Fe and Ni content of the ash of the 3-year combinations.

The application of this physical factor (depending on the duration of exposure to He–Ne laser light) can contribute to obtaining ash richer in macro- and microelements which can be used as eco-friendly plant fertilizer characterized by lower-impact production than commercial fertilizers. The pre-sowing stimulation of seeds with He–Ne laser light is a way to influence the chemical composition of *Lavatera* ash. Further studies including other plants are needed to confirm the observed relationships.

## Data Availability

The datasets generated and analysed during the current study are available from the corresponding author on reasonable request.
